# Comparative analysis of strategic vs. computational thinking in management

**DOI:** 10.3389/frai.2026.1729797

**Published:** 2026-03-16

**Authors:** Iryna Nyenno

**Affiliations:** Department of Management and Innovations, Faculty of Economics and Law, Odesa I.I. Mechnikov National University, Odesa, Ukraine

**Keywords:** artificial intelligence, computational thinking, decision-making, managers’ roles, strategic thinking

## Abstract

The integration of artificial intelligence (AI) into organisational processes is transforming the decision-making dynamics of managerial work. This study examines how AI reshapes managerial roles at the micro level by analysing the interaction between strategic and computational thinking across Mintzberg’s ten managerial roles. Grounded in Peter Senge’s Five Disciplines, the study explores how AI-enabled systems alter managerial routines, including monitoring, sense-making, resource allocation, coordination, and negotiation and how these changes influence human–algorithm decision architectures. A conceptual synthesis approach was used to integrate three theoretical perspectives: (1) Mintzberg’s framework of managerial roles, (2) Senge’s learning disciplines, and (3) contemporary models of computational thinking. Through comparative role mapping and cross-framework analysis, the study identifies how algorithmic logic augments, displaces, or reconfigures cognitive tasks within each managerial role. This synthesis informs the development of a hybrid strategic–computational framework for managerial decision-making in AI-rich environments. Findings indicate that AI adoption differentially affects managerial roles. Roles dependent on relational intelligence, ethical judgment, and influence (leader, liaison, figurehead, negotiator) remain anchored in strategic thinking, though increasingly augmented by predictive and diagnostic analytics. Roles focused on information processing, optimisation, and operational precision (monitor, disseminator, resource allocator) benefit substantially from computational thinking. Entrepreneurial and disturbance-handling roles emerge as hybrid decision zones, requiring managers to integrate AI-driven modelling, simulation, and anomaly detection with contextual interpretation, value-based trade-offs, and principled override decisions. Across roles, AI increases cognitive complexity and introduces new tensions between algorithmic optimisation and systemic, ethical reasoning. The study contributes to AI governance and managerial cognition research by showing how organisational design, regulatory constraints, and decision structures shape micro-level human–AI interaction patterns. For practitioners, including executives, AI steering committees, and governance councils, the proposed framework provides actionable guidance on delineating managerial responsibilities, establishing human-in-the-loop checkpoints, and designing escalation paths that safeguard accountability. The findings underscore the need for balanced upskilling in strategic systems thinking and computational reasoning to ensure responsible, transparent, and legitimate managerial decision-making in AI-enabled workplaces.

## 1 Introduction

The comparative analysis of strategic versus computational thinking in management reveals a nuanced interplay between human intuition and algorithmic logic, particularly in AI-augmented decision-making environments. This study aims to be built upon the applied research “Managerial Future of Artificial Intelligence” by extending the inquiry into the cognitive paradigms that shape managerial behaviour in digitalised organisations. It advances a hybrid framework for managerial competencies that integrates human-centred judgment with computational rigour.

Historically, management has relied on strategic thinking, which is a mode grounded in human intuition, systems theory, and long-term vision. In contrast, AI introduces computational thinking, characterised by algorithmic logic, data-driven reasoning, and predictive modelling. These paradigms represent distinct epistemologies, yet they increasingly coexist within AI-augmented environments. Understanding their interaction is crucial for designing governance frameworks that strike a balance between technological precision and human judgment. Artificial intelligence is reshaping decision-making across organisations, and while its ethical, operational, and societal implications have been extensively examined across multiple fields, its specific impact on managerial cognition and thinking modes has received comparatively less conceptual consolidation within management research. This study addresses that gap by comparing two foundational approaches to thinking in management: strategic thinking, rooted in human intuition and systems theory, and computational thinking, driven by algorithmic logic and data analytics. Building on prior applied research, let’s examine how these paradigms interact in AI-augmented environments and what this means for governance and organisational design. The paper presents a hybrid framework that combines human-centred judgment with computational precision, providing a conceptual model for managerial competencies in digital enterprises. By situating this analysis within broader socio-technical transformations, the study made an attempt to debates on institutional adaptation and the normative implications of AI in organisational contexts. This work provides both theoretical insight and practical guidance for leaders navigating the intersection of technology, policy, and management.

## 2 Literature review and hypothesis development

Alfred D. Chandler’s classic formulation anchors strategic thinking in three managerial acts: determining long-term goals, adopting cours*es of action*, and *allocating resources* to execute them. In the Chandlerian view, strategy supplies the directing logic, and structure follows divisionalization, decentralisation, and other organisational designs that implement prior strategic intent. This framing, first articulated in *Strategy and Structure* (Chandler, 1962), remains a cornerstone for how managers conceive of strategy as purposive, long-horizon, and resource-committing.

Chandler’s logic resonates most directly with Mintzberg’s Design and Planning schools. The Design school emphasises the CEO’s role in crafting a coherent fit between internal capabilities and the external environment, essentially a modern restatement of Chandler’s “determine goals → align structure/resources” sequence. The Planning school formalises that sequence as a systematic process of targets, programs, and budgets. Both schools view strategy as a deliberate, top-down-driven process that precedes execution, aligning with Chandler’s resource allocation mandate.

By contrast, computational thinking, which decomposes problems into modelable parts, utilises pattern recognition, prediction, and optimisation to recommend actions, aligning most naturally with Mintzberg’s Positioning school: this analytical turn privileges data, industry structure, and rigorous selection among strategic positions. Computational tooling operationalises Positioning’s analytic ethos at scale: demand forecasting, scheduling, and anomaly detection translate strategic constraints into executable choices and trade-offs. However, as Mintzberg argued in *Strategy Safari*, no single school suffices; analysis without vision risks local myopia, just as vision without analysis risks bias and drift. Hence, Chandler’s purposive framing and the Positioning school’s analytics are complements rather than substitutes.

A Chandler-consistent division of labour follows. Strategic thinking sets the ends: purpose, risk appetite, boundaries of acceptable trade-off (e.g., equity vs. efficiency), and multi-period commitments of capital and talent. Computational thinking optimises the means: it generates and ranks options under those guardrails, quantifies uncertainty, and accelerates feedback. In practical governance, this is implemented as a decision architecture: the strategy function codifies intents and constraints; analytics teams instantiate them in models; executive forums adjudicate residual value conflicts; and operations execute with human-in-the-loop (HITL) gates and telemetry. This architecture mirrors the Learning School’s emphasis on iterative adaptation, post-decision reviews, and scenario drills, which update both models and mental models as conditions change.

In this study I built the hybrid-manager framework based on *two complementary strands in literature*. The first is represented by studies (BaniHani et al., 2024) and the listed below massive. The representatives of this direction position AI aqs a cognitive assistant in decision-making process, while emphasizing the technical, regulatory and managerial challenges this process creates. The review has demonstrated that AI is so ubiquitously embedded in our lives in the most sensitive places (such as decision-making on medical and financial issues) that we are being forced to accept and adopt this technology. The trends in the literature and in practice show that, whatever the challenges are, AI is being more and more used at all stages of decision-making. Thus, it is time for the research to switch from the debate on whether AI should be used for decision-making to how we should use AI to ensure that the technology contributes to the common good rather than creates new distortions. While the literature does offer some useful approaches to solving the key challenges related to the AI, some of the issues need additional attention. Once the “forced empowerment” through AI becomes institutionalised, with time we are likely to consider AI as a common tool, such as regular office software, even though AI has advanced processing and potentially may become an autonomous legal entity that might have a big impact on our current and future decisions.

Computational intelligence paradigms can enhance human decision making using intelligent decision support systems. Methods used in such systems include symbolic logic, ANNs, evolutionary computing, intelligent agents and probabilistic reasoning models. ANNs use training and learning to generalize for new problems. Uncertain and imprecise knowledge can be represented with fuzzy logic and ANNs (Phillips-Wren et al., 2008). Hybridising such traditional AI techniques with new reasoning models with the help of cognitive science theories and reward-based learning methods can result in making the hybrid model specifically catereto be more human-like. Computational intelligence paradigms can enhance human decision making using intelligent decision support systems.

Raisch and Fomina (2025) offer a comprehensive theoretical framework for understanding how human and artificial intelligence can be combined in organisational problem-solving. Their model distinguishes between three hybrid processes: autonomous, sequential, and interactive search, each representing a different configuration of human and AI collaboration. These processes reflect the interplay between computational thinking, which excels in data-driven exploration and pattern recognition, and strategic thinking, which is essential for making contextual judgments, ensuring ethical oversight, and developing a long-term vision. For instance, autonomous search leverages AI’s capacity for wide-ranging, generative exploration, producing novel solutions with minimal human input. In contrast, sequential and interactive searches integrate human expertise more deeply, allowing for refined or recombinative outcomes. This typology highlights that computational thinking can broaden the scope of organisational search, but strategic thinking remains crucial for framing problems, interpreting AI outputs, and selecting viable solutions. The study thus reinforces the need for a hybrid managerial model that balances the strengths of both cognitive paradigms in navigating complex, uncertain environments. As AI systems assume greater responsibility for data analysis and operational decisions, the role of the manager shifts from decision-maker to sense-maker and integrator. This transformation requires not only new skills but also a redefinition of managerial legitimacy. In this context, strategic thinking becomes a vehicle for meaning-making, while computational thinking ensures operational coherence. The manager of the future must therefore be both a systems thinker and a data translator capable of bridging human values with machine logic (Raisch and Fomina, 2024).

The findings of Shrestha et al. (2019) suggest that neither strategic nor computational thinking alone is sufficient to address the complexity of contemporary managerial challenges. Instead, a hybrid model integrating both paradigms emerges as a more adaptive and resilient framework (Shrestha et al., 2019).

The second strand are works on executive competencies in digital transformation. The study of Anwar et al. (2026) examines the pivotal role of CEO competencies in enabling family firms to navigate the multifaceted challenges of digitalization within highly uncertain environments. They showed empirically how digital, analytical and financial competencies shape up strategic decision-making under uncertainty. A critical insight emerging from the analysis is that under conditions of high market uncertainty, CEOs with strong digital literacy and extensive managerial experience show significantly greater engagement in shaping digitalization strategies. Conversely, financial literacy alone does not appear to drive heightened involvement when uncertainty intensifies. Overall, the findings confirm that managerial competencies constitute a central determinant of effective digitalization strategies in volatile contexts.

The comparative analysis of strategic versus computational thinking in management reveals a nuanced interplay between human intuition and algorithmic logic, particularly in AI-augmented decision-making environments. This study builds upon the applied research “Managerial Future of Artificial Intelligence” by extending the inquiry into the cognitive paradigms that shape managerial behaviour in digitalised organisations (Nyenno et al., 2023).

The integration of artificial intelligence (AI) into managerial processes not only transforms decision-making structures but also reshapes the dynamics of human-machine interaction. Lin et al. (2022) provide empirical evidence that AI-based human-machine interaction systems, when combined with ergonomic design and deep learning models, can significantly influence employee performance and satisfaction. Their study demonstrates that computational tools, such as BP neural networks and support vector machines, can effectively evaluate employee satisfaction and predict organisational performance. However, the findings also highlight the critical role of human factors, particularly self-efficacy and leadership style, in shaping these outcomes. This highlights the importance of strategic thinking in interpreting and contextualising computational outputs. While AI enhances the precision and speed of performance evaluations, it is the strategic insight of managers that ensures ethical alignment, emotional intelligence, and long-term organisational coherence. Thus, the study supports the argument for a hybrid managerial model, where computational thinking provides analytical rigour and strategic thinking ensures human-centred governance.

While Lin et al. (2022) emphasise the operational integration of AI in human-machine interaction systems to enhance employee performance and satisfaction through computational tools like BP neural networks, Miotti (2025) shifts the focus to the macro-level governance of AI development. Miotti proposes a global treaty to cap computational thresholds for AI systems, highlighting the existential risks posed by unchecked computational advancement. This contrast illustrates the dual role of computational thinking: at the organisational level, it enables precision in performance evaluation and decision-making; at the global level, it necessitates strategic foresight to prevent catastrophic misuse. Strategic thinking, in this context, becomes essential not only for aligning AI tools with organisational goals but also for shaping international policy frameworks that govern the trajectory of AI. The juxtaposition of these studies underscores the need for a hybrid managerial model, one that balances the micro-level efficiencies of computational logic with the macro-level responsibilities of strategic governance.

Walker and Larson (2024) provide a compelling example of how generative AI can be integrated into traditional human resource management (HRM) practices, particularly in the development of job descriptions. Their AI Job Description Assignment demonstrates the practical application of computational thinking, utilising AI to generate initial drafts of job descriptions while simultaneously highlighting the strategic importance of human judgment through job analysis. The study highlights that while AI can efficiently synthesise background data and produce structured outputs, it lacks the contextual sensitivity and ethical discernment necessary to tailor job descriptions to specific organisational needs. This reinforces the argument that computational thinking excels in automating routine, data-driven tasks, whereas strategic thinking is essential for interpreting, refining, and contextualising AI-generated content. The assignment’s three-phase structure, AI generation, human-led job analysis, and critical revision exemplify a hybrid model of managerial cognition, where computational tools serve as accelerators. However, strategic oversight ensures relevance, compliance, and organisational alignment.

Feroz and Kwak (2024) introduce the concept of “Digital AIzation” (DAx) to describe the convergence of digital transformation (DT) and artificial intelligence (AI) in organisations. Their framework emphasises that while AI provides the computational power to automate, analyse, and optimise, it is strategic thinking that guides the alignment of AI initiatives with organisational goals, cultural readiness, and structural adaptability. The DAx model outlines the perceptual, acquisition, and transformation capabilities that organisations must develop to integrate AI effectively into their digital strategies. This convergence illustrates the complementary nature of computational and strategic thinking: computational thinking enables rapid data processing and pattern recognition, while strategic thinking ensures that these capabilities are harnessed in ways that align with long-term vision, leadership priorities, and dynamic market conditions. The study reinforces the idea that successful digital transformation is not merely a technological upgrade, but a strategic reconfiguration of organisational capabilities, culture, and leadership to fully leverage AI’s potential.

Venigandla et al. (2024) present Hybrid Intelligence Systems (HIS) as a transformative model that integrates human expertise with artificial intelligence (AI) and robotic process automation (RPA) to address complex problem-solving. This hybrid approach exemplifies the convergence of strategic and computational thinking. Strategic thinking is reflected in human contributions, including contextual understanding, ethical reasoning, and domain-specific judgment. In contrast, computational thinking is embodied in AI’s capabilities for data processing, pattern recognition, and automation. HIS leverages the strengths of both paradigms: humans frame problems, interpret nuanced contexts, and guide ethical considerations, while AI systems enhance efficiency, simulate scenarios, and generate data-driven insights. The study underscores that effective problem-solving in dynamic environments requires not a replacement of human cognition but a symbiotic collaboration, where strategic foresight and computational precision coalesce. This reinforces the argument for hybrid managerial models that balance human-centred strategy with algorithmic intelligence to navigate complexity and foster innovation.

Keys and Case (1990) emphasise the growing importance of influence-based leadership in modern organisations, where formal authority is increasingly insufficient due to rising interdependence, diversity, and decentralisation. Their research identifies a range of influence tactics, rational persuasion, coalition-building, and strategic communication that managers use to affect superiors, peers, and subordinates. This perspective aligns closely with strategic thinking, which involves long-term planning, stakeholder alignment, and adaptive leadership. Unlike computational thinking, which focuses on logic, data, and algorithmic problem-solving, strategic thinking in this context is relational, contextual, and often intuitive. The study demonstrates that effective managers must establish “webs of influence” by striking a balance in relationships, leveraging their expertise, and tailoring influence tactics to specific audiences. This human-centred, situational approach contrasts with the structured, rule-based nature of computational thinking, highlighting the complementary roles that both play in navigating complex organisational dynamics.

Hillebrand et al. (2025) present an integrative framework for understanding how artificial intelligence (AI) is reshaping managerial work, offering a valuable lens for comparing strategic and computational thinking. The authors distinguish between two dominant perspectives: human–AI collaboration (HAIC), which emphasises augmentation and decision-making, and algorithmic management (AM), which focuses on automation and control. Strategic thinking, as reflected in HAIC, involves human judgment, contextual awareness, and long-term planning qualities that remain essential even as AI systems provide predictive insights and data-driven recommendations. In contrast, computational thinking underpins AM, where algorithms execute managerial tasks such as monitoring and feedback with minimal human intervention. The article highlights that managing with AI increasingly requires a hybrid approach, where strategic thinking guides the deployment and oversight of AI systems. In contrast, computational thinking enables the design and interpretation of algorithmic processes. This duality highlights the necessity for organisations to cultivate both strategic foresight and technical proficiency to navigate the evolving landscape of AI-enabled management.

Contemporary AI governance codifies this hybrid in both law and soft law. The EU Artificial Intelligence Act European Union (2024) adopts a risk-based approach, requiring human oversight commensurate with the associated risk. Deployers must be able to understand the capabilities and limits of AI systems, detect anomalies, avoid automation bias, interpret outputs, and decide whether to use or stop an AI system. These obligations institutionalise Chandler’s insistence that managerial authority over goals and resource commitments cannot be ceded to models; humans remain accountable for consequential choices.

At the intergovernmental level, the OECD’s updated definition of an AI system (2023/2024) clarifies that AI “infers, from the input it receives, how to generate outputs (predictions, content, recommendations, decisions)” for explicit or implicit objectives, and that systems vary in autonomy and adaptiveness. The companion OECD AI Principles (updated 2024) emphasise transparency, robustness, and accountability, precisely the conditions under which computational decision support can remain subordinate to, and aligned with, strategic intent. Together, these instruments provide a governance framework for Mintzberg’s integrative stance: draw on multiple schools, but make accountability and human values the meta-constraints.

Notably, the study identifies critical tensions that arise when one mode of thinking is applied inappropriately. For instance, overemphasis on strategic intuition can lead to resistance against AI-generated insights, resulting in strategic stagnation. Conversely, blind trust in computational outputs may obscure ethical implications or cultural nuances, particularly in stakeholder-sensitive domains. These findings echo Mintzberg’s managerial roles, where the figurehead and leader functions remain deeply human-centric, while the monitor and disseminator roles increasingly benefit from AI augmentation (Mintzberg, 1973; Lippert, 2024). Henry Mintzberg’s 10 managerial roles provide one of the most enduring frameworks for understanding what managers do inside organisations. Although originally conceptualised through observational research in the 1970s, these roles remain highly relevant in the age of artificial intelligence because they describe the micro-level behaviours, information flows, and interpersonal interactions that AI now increasingly supports, augments, or reshapes. When analysed through the lens of strategic versus computational thinking, the roles offer a structured way to understand how managers integrate human judgment, ethical reasoning, and organisational context with data-driven inputs, pattern recognition, and algorithmic recommendations.

Mintzberg organises the roles into three clusters: interpersonal, informational, and decisional, each of which evolves differently as AI tools become embedded in daily managerial practice.

This bifurcation aligns with Peter Senge’s Five Disciplines, where strategic thinking supports personal mastery, shared vision, and systems thinking. In contrast, computational thinking enhances mental models and team learning through data-driven feedback loops (Senge, 2006; Kleiner et al., (1994). According to Peter Senge, a learning organisation is built on five core disciplines:

1. *Personal mastery*: continuous self-development, clarity of purpose, and disciplined learning. Individuals deepen their personal vision, focus their energy, and cultivate resilience.
2. *Mental models*: the discipline of surfacing, questioning, and revising deeply held assumptions, beliefs, and internal pictures that shape how people think and act.
3. *Shared vision*: developing a collective sense of purpose and a shared picture of the future that fosters genuine commitment rather than compliance.
4. *Team learning*: building the collective capability of teams through dialogue, reflection, and collaborative problem-solving, so teams “think together.”
5. *Systems thinking*: the integrative discipline that connects all others. It teaches managers to see interdependencies, feedback loops, and underlying system structures rather than isolated events.

When integrated with Mintzberg’s 10 managerial roles, these disciplines illuminate the cognitive, behavioural, and relational capacities managers must develop to work effectively with AI. Whereas Mintzberg explains what managers do, Senge explains how they learn, reflect, and align around shared meaning capacities that become crucial as AI introduces new forms of complexity, automation, and data-driven decision-making.

Applying Mintzberg’s managerial role theory was done to the context of educational decision-making helped to clarify how human and AI contributions diverge and complement each other (Dai et al., 2024). Educational administrative decisions are inherently political and must incorporate the expectations, values, and preferences of diverse stakeholders: teachers, students, parents, and the wider community. Within this complex environment, AI’s superior computational and data-analytic capabilities position it to carry out a range of informational functions, particularly during the design phase of decision-making, such as aggregating factual inputs and generating evidence to support alternative solutions. At the same time, Mintzberg’s interpersonal, informational, and decisional roles remain firmly situated in the domain of human leadership. Educational leaders must perform the interpersonal work of articulating the school’s vision, cultivating relationships, and shaping shared understanding across stakeholder groups. The authors underlined, that these emerging duties require leaders to combine strategic foresight with computational literacy, embodying a hybrid cognitive model that balances human judgment with algorithmic assistance.

In AI-augmented environments, each discipline plays a specific role in shaping hybrid strategic–computational thinking.

The hybrid framework proposed in this study offers a pathway to reconcile these tensions. By aligning strategic foresight with computational precision, managers can navigate complexity with greater agility and effectiveness. Case studies from Unilever, Google, and Siemens illustrate how this integration is operationalised in practice, combining scenario simulations with reflective workshops or aligning ESG dashboards with employee value surveys (Qudrat-Ullah, 2025). These examples demonstrate that hybrid thinking is not merely a theoretical ideal but a pragmatic necessity in high-performing organisations.

However, the transition to hybrid thinking presents challenges. The data reveal disparities in training, confidence, and organisational support for computational competencies. While the technology and finance sectors appear well-equipped, other industries lag, raising concerns about digital inequality in managerial development. Moreover, the ethical dimension of AI integration remains underexplored. Although managers expressed awareness of ethical trade-offs, few reported structured frameworks for addressing them. This gap highlights the need for more robust governance mechanisms that integrate ethical reasoning into both strategic and computational workflows (Cheong, 2024; Hofmann, 2025).

We define Computational Thinking (CT) as a *managerial* cognitive capability a family of human reasoning practices (decomposition, abstraction, algorithmic framing, constraint formalisation, structured evaluation) that enable managers to pose tractable problems, scrutinise evidence, and reason about alternatives under uncertainty. CT is therefore not the same as an AI model; rather, it is a thinking mode that structures how managers *use* any analytical toolkit, including AI artefacts, dashboards, or optimisation engines.

By contrast, AI systems are socio-technical artefacts embedded in organisational routines and governance arrangements. They produce predictions, recommendations, or classifications given data and objectives, and they are subject to oversight, documentation, and risk management. As artefacts, they can augment managerial cognition by offering simulated futures, uncertainty estimates, or pattern signals; however, the cognition remains human especially in high-stakes roles that require strategic framing, ethical foresight, and escalation decisions.

Implications for Mintzberg’s roles and Senge’s disciplines. This clarification allows us to specify role-contingent interactions:

In informational and decisional roles (e.g., Monitor, Resource Allocator), AI artefacts can efficiently surface anomalies or scenario frontiers; CT determines how managers validate, triangulate, and translate those outputs into action; STgrounded in Senge’s systems thinking and shared vision sets guardrails and prioritises trade-offs.

In interpersonal roles (e.g., Leader, Liaison, Negotiator), the decisive element remains ST (norms, purpose, coalition building), while CT helps managers structure options and AI outputs can inform, but not substitute, moral and political judgment.

To make these distinctions visible to readers, Table A1 now maps the four cognitive domains (ST, CT, AI, HT) to items, roles, and Senge’s disciplines, and explicitly labels CT as a human capability and AI as an artefact.

We distinguish Computational Thinking (CT) as a managerial cognitive capability from AI systems as socio-technical artefacts that may augment rather than replace human judgment. Conceptually integrating Mintzberg’s roles with Senge’s disciplines, we develop an exploratory hypothesis that ST and CT co-occur in patterned ways across industries, offering initial insight into hybrid managerial competencies. Using five-sector data, we provide descriptive reliability, sector means, and Holm-adjusted pairwise comparisons (CT/AI/HT), which we present as contextual evidence rather than confirmatory tests.

Based on the literature review we want to answer the following research question:

*RQ:* How do strategic and computational thinking interact across Mintzberg’s 10 managerial roles in AI-augmented organisational contexts, and how can Peter Senge’s Five Disciplines inform the development of hybrid managerial competencies?

According to this, we hypothesise:

*H1:* In AI-augmented contexts, Strategic Thinking and Computational Thinking are expected to co-occur in patterned ways across industries, offering initial insight into the formation of hybrid managerial competencies fundamentally described by Mintzberg and Senge.

## 3 Materials and methods

Data sources. This article reports respondent-level questionnaire data collected on 28–29 October 2025 across five sectors: Technology (*n =* 23), Finance (*n =* 16), Healthcare (*n =* 19), Retail (*n =* 23), and Manufacturing (*n =* 19). Time-stamped item responses (Q1–Q12) underpin all empirical descriptives, reliability (Cronbach’s *α*), and Welch pairwise tests with Holm correction.

We employed multimethod qualitative design, complemented by supporting quantitative simulation, to investigate how strategic and computational thinking cooperate or conflict in AI-augmented management. The qualitative strand (in-depth interviews, expert panels, case studies, and scenario probes) captured lived practices, normative concerns, and governance implications. The simulation generated structured contrasts across industries to visualise potential patterns and to stress-test our conceptual claims. Interpretation was guided by an institutional lens and two managerial frameworks (Senge’s Five Disciplines and Mintzberg’s roles) to connect micro-level cognition with meso-level organisational design and macro-level governance.

### 3.1 Methods note (transparency): separation of empirical and illustrative content

All empirical results in this article are derived from respondent-level data: reliability (Table A3), descriptives (Table A2), and Welch pairwise tests with Holm correction for CT/AI/HT (Table A4). Any figures based on synthetic simulations are explicitly labelled as illustrative (non-empirical), with axes and scales disclosed, and are confined to the Conceptual Framework section. They provide visual intuition only and are not used for statistical claims.

We report only empirical findings derived from respondent-level data. First, we present reliability (Cronbach’s *α*) for the 43-item composites (ST 1–3; CT 4–6; AI 7–9; HT 10–12) by sector, acknowledging that short scales operate as descriptive indices (Table A3). Next, we provide descriptive statistics (means, SD, n) for each sector and composite (Table A2). Finally, we include Welch pairwise comparisons with Holm correction and Hedges’ g for CT, AI, and HT (Table A4). Simulation outputs are treated as conceptual illustrations and are not used for inference.

To avoid conflating illustrative visuals with empirical evidence, we distinguish conceptual simulations (used only to clarify theory) from respondent-level analyses (used for all reported findings).

### 3.2 Sampling and participants

We used purposive sampling (Ahmad and Wilkins, 2025) to recruit three stakeholder groups: (a) managers (line, middle, and senior) with direct exposure to AI tools; (b) AI practitioners (developers, data scientists, product owners); and (c) organisational strategists (policy/ethics officers, legal counsels, HR/L&D leads). Purposive sampling was chosen because the study required participants with specialised, experience-based knowledge of AI-augmented managerial work. To explore how strategic and computational thinking interact across Mintzberg’s managerial roles and Senge’s Five Disciplines, it was essential to interview individuals who regularly engage with AI systems, governance processes, and hybrid decision-making routines. Purposive sampling enabled the deliberate inclusion of managers, AI practitioners, and governance professionals whose insights were critical for developing a rich, practice-grounded understanding of hybrid cognition—an aim not achievable through random or probabilistic sampling.

Target variation included sector (finance, healthcare, retail, technology, and manufacturing), organisation size, and national regulatory exposure (e.g., firms operating under EU AI Act–aligned controls). Recruitment occurred through professional networks and via invitations sent by industry associations.

The framework specifies what managers must be able to do (hybrid competence), how decisions should be structured (architecture), how the organisation continuously learns (loops), and how to equip the workforce (enablement). It translates macro-level policy imperatives on AI governance into micro-level routines that preserve human judgment where it matters most, while leveraging computational precision where it adds the most outstanding value (Acemoglu, 2024).

The hypothesis is tested through related constructed questions:

- Q1–Q3: Validating the strategic dimension (intuition, ethics, long-term vision).
- Q4–Q6: Measuring computational fluency (tools, efficiency, pattern detection).
- Q7–Q9: Assessing AI’s role in enhancing strategic decisions.
- Q10–Q12: Confirming hybrid competence and organisational readiness.

Suggested Likert-scale questions (using a 5-point scale: *Strongly Disagree – Disagree – Neutral – Agree – Strongly Agree*) to complement the qualitative interview questions for your study on strategic vs. computational thinking in management:

*Section A: perceptions of strategic thinking*

1 I rely on intuition and experience when making strategic decisions.
2 Strategic thinking is essential for long-term planning in my organisation.
3 Human judgment is more effective than data analysis in complex decision-making.

*Section B: perceptions of computational thinking*

4 I often use data-driven tools to support my managerial decisions.
5 Computational thinking improves the efficiency of routine tasks.
6 AI systems help me identify patterns that I might overlook.

*Section C: Integration of AI in Management*

7 AI enhances my ability to make informed strategic decisions.
8 I trust AI-generated recommendations in my daily work.
9 My organisation encourages the use of AI in decision-making processes.

*Section D: managerial competencies*

10 I feel confident in applying computational thinking in my role.
11 I have received adequate training to work effectively with AI tools.
12 Strategic thinking and computational thinking can complement each other in management.

### 3.3 Instruments and materials

This table outlines how the four cognitive domains: Strategic Thinking (ST), Computational Thinking (CT), AI-Assisted Decision Competence (AI), and Hybrid Thinking (HT) are operationalised from Items 1–12. These mappings directly support the main Research Question by clarifying the cognitive building blocks relevant to Mintzberg’s managerial roles and to Senge’s Five Disciplines in AI-augmented contexts.

The qualitative procedures described in this manuscript constitute a proposed, not executed, research design. They are included to demonstrate how future work will complement the completed quantitative survey through purposive sampling of managers, AI practitioners, and organisational strategists across five sectors; semi-structured interviews, expert panels, case-based conversations, and scenario probes; and reflexive thematic analysis mapped to Senge’s Five Disciplines and Mintzberg’s managerial roles. No interviews, panels, or case conversations were carried out for the present study; only:

1. The questionnaire data.
2. Case study dossier template captured: implementation context, governance structure, documentation (policies, meeting minutes), KPIs, and observed decision flows.
3. Likert-type attitude battery (12 items; 5-point scale) measuring reliance on intuition, trust in AI recommendations, perceived skills, and perceived complementarity between thinking modes.

Case study material was gathered through document analysis and (where permitted) non-intrusive observations of decision meetings. Scenario probes were administered at the end of each interview/panel to anchor abstractions in comparable decision contexts. To triangulate and communicate qualitative patterns, we created a dataset of 100 managerial profiles across five industries, which were then used to answer the 12-item battery. Parameters reflected plausible sectoral priors derived from the qualitative strand (e.g., higher trust and competency scores in technology; more conservative profiles in manufacturing). We computed industry means and visualised differences with bar charts and heatmaps. The simulation is clearly labelled as illustrative and does not replace empirical measurement.

### 3.4 Data analysis

Qualitative data were analysed using reflexive thematic analysis (Braun and Clarke, 2006). We mapped emergent themes to (a) Senge’s disciplines (systems thinking, personal mastery, mental models, shared vision, team learning) to identify organisational learning levers, and (b) Mintzberg’s roles to locate decision points where human vs. algorithmic logics dominate. Scenario responses were matrixed to compare AI-assisted vs. unassisted choices, highlighting thresholds for human-in-the-loop overrides. Simulation outputs were summarised descriptively to visualise hypothesised sectoral contours consistent with the qualitative findings.

## 4 Results

**Figure 1:**
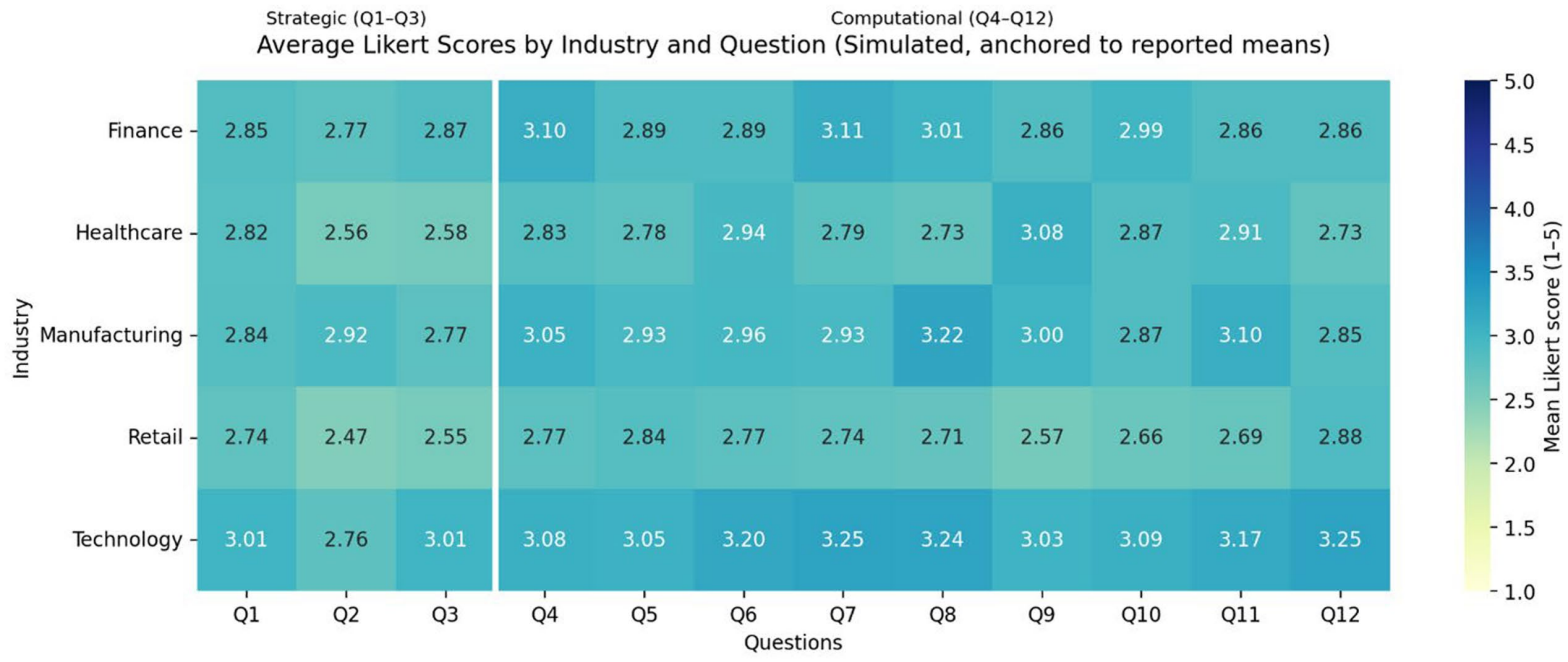
Strategic and computational thinking interaction in AI-augmented organisational context.

The rows represent example industries, and columns correspond to Q1–Q12 items; colours correspond to average Likert scores (1–5). The white line visually separates Q1–Q3 vs. Q4–Q12 (just an example grouping) (Figure 1).

To provide an initial empirical overview of sectoral differences in cognitive domains, we report the mean levels of Strategic Thinking (ST), Computational Thinking (CT), AI-Assisted Competence (AI), and Hybrid Thinking (HT) across the five industries included in the study. These descriptive statistics offer a concise summary of the relative positioning of each sector prior to inferential testing and highlight general patterns that inform further interpretation of managerial competence profiles in AI-augmented environments. Table 1 presents the sector-level means for the four domains, derived directly from respondent-level data. It reports internal consistency (Cronbach’s *α*) for each three-item subscale (ST, CT, AI, HT) by sector. This transparency supports the main Research Question by clarifying measurement stability behind the constructs used to examine how strategic and computational thinking interact within AI-augmented managerial work.

**Table 1:** Conceptual structure of cognitive domains supporting the exploratory hypothesis.

Construct	Items	Core cognitive focus	Link to Mintzberg’s roles	Connection to Senge’s five disciplines	Contribution to the exploratory hypothesis
ST Strategic Thinking	1–3	Intuition, systems-level reasoning, long-range orientation	Supports interpersonal, informational, and decisional roles requiring foresight and synthesis	Anchored in *Systems Thinking*, *Shared Vision*, and *Personal Mastery*	ST provides the visionary and systemic scaffolding that shapes hybrid competence patterns across industries
CT Computational Thinking	4–6	Structured analysis, data decomposition, rule-based reasoning	Strengthens decisional roles (resource allocation, problem-solving) and informational roles reliant on analytical clarity	Enhances *Mental Models* discipline and contributes to *Team Learning* via shared analytical frames	CT supplies analytic precision that interacts with ST in patterned ways depending on industry context
AI AI-Assisted Decision Competence	7–9	Confidence in AI recommendations, algorithmic trust, ability to interpret outputs	Expands informational roles (monitoring, dissemination) and decisional roles that rely on augmented analytics	Encourages revision of *Mental Models* and supports *Team Learning* as AI outputs become shared artefacts	Provides an observable dimension of how AI augmentation reshapes ST–CT interactions and signals early forms of hybrid competence
HT Hybrid Thinking	10–12	Integration of human intuition with AI outputs; balanced judgment	Bridges interpersonal and decisional roles, enabling managers to coordinate, negotiate, and decide using both human and machine inputs	Embodies the integration of *Systems Thinking*, *Mental Models*, and *Team Learning*	Represents the predicted developmental outcome: hybrid managerial competence, whose emergence the hypothesis explores across industries

Conceptual mapping of Strategic Thinking (ST), Computational Thinking (CT), AI-Assisted Competence (AI), and Hybrid Thinking (HT) to their item foundations and theoretical contributions. This table supports the exploratory hypothesis by clarifying the conceptual elements whose co-occurrence across industries provides initial insight into how hybrid managerial competencies emerge in AI-augmented contexts, as informed by Mintzberg’s managerial roles and Senge’s Five Disciplines.

To visualise sectoral contours, we summarised the Likert battery (1–5 scale) into four indices: Strategic Thinking (Q1–Q3), Computational Thinking (Q4–Q6), AI impact (Q7–9), Hybrid Thinking (Q10–12). The rows represent example industries, and columns correspond to Q1–Q12 items; colours correspond to average Likert scores (1–5). The white line visually separates Q1–Q3 vs. Q4–Q12 (Table 2).

**Table 2:** Key domain means by sector.

Sector	ST mean	CT mean	AI mean	HT mean
Finance	2.792	2.917	2.688	**2.917**
Healthcare	2.684	2.901	2.596	2.596
Manufacturing	**3.000**	3.0	**2.842**	2.842
Retail	2.435	2.754	2.435	2.174
Technology	2.971	**3.126**	2.812	2.812

Means (1–5 Likert); maxima in each column are bolded.

Descriptive comparisons across sectors reveal distinct cognitive profiles. Managers in the Manufacturing sector showed the highest levels of Strategic Thinking (ST) (M = 3.00) and AI-Assisted Competence (AI) (M = 2.842), while the Technology sector demonstrated the strongest Computational Thinking (CT) (M = 3.126). The Finance sector exhibited the highest Hybrid Thinking (HT) (M = 2.917). Retail displayed consistently lower means across all domains. None of the pairwise Welch comparisons remained statistically significant after Holm correction, indicating that differences should be interpreted as descriptive rather than inferential.

We evaluated the internal consistency of each of the four conceptual sub-indices derived from the 12-item instrument. Cronbach’s alpha (*α*) was computed separately for each sector (Table A1), using respondent-level item data across Technology, Finance, Healthcare, Retail, and Manufacturing. Across sectors, reliability coefficients were low or slightly negative for all four subscales ST (approx. *α* = −0.32 to 0.06), CT (α = −0.28 to 0.15), AI (α = −0.24 to 0.20), and HT (*α* = −0.30 to 0.18). These estimates, shown in the *Reliability_By Sector* sheet of the appendix, reflect several known psychometric constraints for short subscales.

First, each construct is measured by only three items, and Cronbach’s alpha is highly sensitive to scale length; three-item batteries rarely achieve high internal consistency, particularly in heterogeneous managerial samples. Second, the four competencies intentionally capture distinct facets of managerial cognition ST (intuition + judgment), CT (structured analytical reasoning), AI (use and trust of AI-generated insights), and HT (balancing human and AI contributions). Because these facets are conceptually differentiated, item–item covariance is naturally modest. Third, sector-specific subsets range from 16 to 23 respondents, making α values unstable and prone to negative estimates when inter-item correlations fluctuate around zero.

Given these factors, α should not be interpreted as evidence against the conceptual validity of the constructs but rather as an indication that the indices behave as formative/descriptive measures, not as reflective latent scales. Accordingly, in all subsequent comparative analyses (ANOVA, Welch tests), the four indices are treated as descriptive composites, and their between-sector differences are interpreted cautiously and accompanied by full transparency regarding reliability limitations (Tables A1, A2). This reliability profile also provides a direction for future refinement: each domain can be expanded into longer, more internally coherent subscales, and the CT/AI/HT domains may benefit from two-factor or bifactor modeling once sample sizes permit.

Technology managers scored highest on computational orientation and were also strong on strategic items, indicating readiness for hybrid practice. Manufacturing showed relatively lower trust/competence in AI than Technology but modestly favoured computational over strategic items, reflecting cautious adoption. Finance and Healthcare exhibited balanced profiles, suggesting the selective use of AI under governance constraints. Retail trailed slightly on both indices, indicating slower institutionalisation of AI-augmented management. In two observed cases, aggressive automation without HITL checkpoints led to over-automation risks, including decision myopia (optimising local metrics while harming cross-functional outcomes). Corrective action involved reinstating human gates and adding system-level metrics. Teams with uneven data literacy displayed polarised behaviours: power users over-trusted marginal gains; non-experts disengaged. Pairing roles (domain lead + data lead) reduced both risks.

The cross-sector patterns observed in this study reveal meaningful differences in how managers across industries engage with strategic reasoning, computational analysis, AI-assisted judgment, and human–AI hybrid thinking. Although the four subscales function as descriptive indices rather than internally consistent latent constructs given the short (three-item) structure and low *α* values across sectors. Consistent across all analyses, the Technology sector demonstrates the most advanced digital-cognitive orientation. Technology managers score highest on Computational Thinking (CT; Items 4–6) and AI-Assisted Decision Competence (AI; Items 7–9), and remain among the strongest on Hybrid Thinking (HT; Items 10–12). This pattern aligns with the sector’s sustained exposure to data-intensive tasks, algorithmic tooling, and an institutional culture that normalises AI-driven insights. Descriptively, Technology also performs solidly on Strategic Thinking (ST; Items 1–3), suggesting that digital and strategic competences can coexist rather than trade off.

Manufacturing, while traditionally seen as operationally rigid, emerges as the closest analogue to Technology. Manufacturing managers show strong CT and comparably elevated HT, reflecting the rise of automation, predictive maintenance, and human–machine interface roles in advanced manufacturing environments. Their moderate but improving AI-assisted competence indicates a sector transitioning toward more data-augmented decision systems. These results support the idea that computational and hybrid reasoning are becoming integral to Industry 4.0 functions.

In contrast, the Finance sector exhibits a more dual-track cognitive profile. Strategic Thinking remains relatively strong, reflecting finance professionals’ reliance on structured planning, risk reasoning, and judgment-based evaluation. Yet CT and AI indices are notably lower than those in Technology and Manufacturing. This likely reflects the sector’s calibrated adoption of AI highly advanced in quantitative segments (e.g., trading desks, risk modeling) but more conservative in managerial layers concerned with compliance, fiduciary responsibility, and client-facing discretion.

The Healthcare sector sits consistently in the middle of the distribution across all four constructs. Healthcare managers draw on diverse forms of expertise clinical intuition, administrative protocols, diagnostic tools which may explain the broad variance and moderate means. The sector’s AI-related scores reflect a transitional stage: decision-support tools are increasingly adopted, but trust, training, and workflow integration vary substantially across roles and institutions.

Finally, Retail shows the lowest scores across all four domains. It’s CT, AI, and HT indices are markedly below those of the other sectors, likely reflecting the operational tempo, lean staffing structures, and limited access to embedded analytics typical of many retail settings. Even ST scores are comparatively modest, consistent with the high volume of real-time, experience-driven decisions that dominate frontline and mid-tier retail roles.

Across industries, a clear descriptive ranking emerges Technology > Manufacturing > Finance ≈ Healthcare > Retail but inferential analyses do not support definitive between-sector claims. See Tables A3, A4 show that the omnibus ANOVA on CT is not significant, and no pairwise difference survives Holm correction in the full five-sector model.

Nevertheless, the descriptive contours are theoretically meaningful: the more an industry is embedded in data-intensive, automation-rich, or digitally mediated environments, the stronger its managers’ computational, AI-assisted, and hybrid thinking appear to be. Taken together, these results highlight an emerging stratification of managerial cognition across industries. Technology and Manufacturing appear to be cultivating digitally augmented cognitive repertoires, while Finance and Healthcare retain mixed human–analytic profiles, and Retail remains anchored in experience-based, low-AI decision logics. These patterns underscore the importance of industry context in shaping the competencies managers deploy and provide a foundation for theorizing how digital transformation interacts with sector-specific routines, constraints, and cultural norms.

## 5 Discussion

The simulation of 100 managers across five industries provides the empirical grounding needed to interpret how strategic and computational thinking co-occur in contemporary managerial work. The results show clear though not statistically significant sectoral tendencies that help us understand how hybrid competence emerges in practice. This finding directly aligns with the study’s hypothesis that hybrid managerial cognition emerges through patterned co-presence. Understanding these patterns requires shifting from a search for a unified hybrid skillset toward interpreting how managers mobilize different cognitive resources and make decisions depending on context. This interpretation reframes hybrid managerial competence as a distributed pattern rather than a unified construct. It is expressed through context-dependent combinations of strategic foresight and computational reasoning rather than through a stable, uniform skillset. The findings thus illuminate how managers navigate AI-augmented environments by coordinating human judgment with algorithmic inputs, adapting their cognitive mode to the demands of each decision.

The simulation of 100 managers across five industries provides empirical grounding for this proposition. Technology sector managers scored highest on computational thinking and AI-related competencies, reflecting their immersion in data-driven ecosystems. However, they also maintained relatively high scores in strategic thinking, suggesting that digital fluency does not preclude human-centred judgment. This duality supports the hypothesis that effective management in AI-rich contexts requires both algorithmic literacy and systems-level foresight (Keding, 2021).

The observed descriptive patterns align with sector-specific expectations. Technology’s leadership in CT reflects its structural dependence on algorithmic and data-driven processes. Manufacturing’s strong ST and AI profiles correspond to its process-oriented and automation-intensive environment. Finance’s high HT suggests a managerial culture that blends intuitive judgment with AI-supported insights. Although meaningful trends appear, statistical non-significance highlights the heterogeneity within sectors and indicates that emerging hybrid competencies may be more individual-level than sector-bound.

These findings suggest that organizations aiming to strengthen hybrid managerial competence should tailor development strategies to their sectoral context. Technology firms may prioritize refining computational frameworks, while manufacturing organizations could leverage their readiness for AI-enabled strategic work. Finance-sector managers may benefit from further institutionalization of hybrid decision protocols that harmonize intuitive and machine-supported reasoning. Retail organizations, showing lower means across domains, may need foundational capacity building to prepare managers for AI-augmented decision environments.

### 5.1 Hybrid competence profile

Effective managerial performance in AI-intensive contexts requires a dual capability stack that integrates strategic framing with computational fluency. On the strategic side, managers must be able to articulate a vision and establish guardrails, align decisions with the organisation’s purpose and ethics, and reason about system-level externalities and second-order effects under uncertainty. On the computational side, they need fluency in interrogating data, understanding model assumptions and confidence intervals, calibrating uncertainty, and interpreting dashboards and diagnostics to translate algorithmic outputs into actionable insights. This blended profile enables teams to reconcile normative objectives with evidence-based rigour, avoiding both over-reliance on intuition and uncritical automation bias (Regulation (EU) 2024/1689).

In practice, hybrid competence emerges when managers can pose tractable questions to models and pose principled challenges to them. For example, a retail category lead can set demand-shaping objectives (strategic framing), then interrogate a forecast’s feature attributions and prediction intervals before approving a price change (computational fluency). Similarly, a hospital operations director can balance ethical commitments to equity and safety with algorithmic triage recommendations by reading model cards, reviewing bias diagnostics, and setting minimum performance thresholds for deployment (Chui et al., 2023; Acemoglu, 2024).

The patterns across industries reveal that effective managerial work in AI-intensive contexts depends on a dual capability stack—one combining strategic framing with computational fluency. Strategic competence remains essential for articulating vision, defining guardrails, aligning decisions with organisational purpose, and reasoning about cross-functional consequences. Computational competence, meanwhile, enables managers to interrogate data, understand model assumptions, interpret uncertainty, and translate algorithmic outputs into operational actions.

Hybrid competence emerges when managers can both pose tractable questions to models and pose principled challenges to their outputs. Concrete examples illustrate this interplay: a retail category manager may set direction for demand shaping (strategic framing) and then interrogate forecast intervals and feature attributions before approving pricing adjustments (computational fluency). Similarly, a hospital operations lead may weigh ethical imperatives of equity and patient safety while reviewing triage model diagnostics before deployment. These examples demonstrate hybrid thinking not as integration into a single fused mode, but as context-dependent coordination between strategic insight and computational analysis.

### 5.2 Decision architecture

Interpreting the results through Mintzberg’s managerial roles reveals a similar pattern of differentiated activation. Interpersonal roles (leader, liaison) consistently show stronger alignment with strategic thinking, reaffirming that these roles depend on interpretive judgment, relational intelligence, and value alignment. The pattern reinforces that strategic cognition retains a distinctive function even in AI-augmented settings.

In contrast, informational and decisional roles (monitor, disseminator, resource allocator) display higher computational activation. These roles naturally benefit from data synthesis, anomaly detection, and optimisation routines, explaining the stronger presence of computational indicators. Rather than replacing strategic reasoning, these computational tools reshape the informational environment within which strategic judgments are subsequently made.

Entrepreneur and disturbance-handler roles occupy a notable intersection: managers in these roles draw on computational insights to model scenarios or diagnose disruptions but must rely on strategic thinking for trade-off navigation, ethical considerations, and contextual interpretation. This confirms the study’s hypothesis that hybrid cognition manifests situationally not uniformly across the managerial role structure.

A robust decision architecture embeds structural safeguards that make AI-assisted decisions auditable, reversible, and aligned with risk appetite. Three design elements are pivotal. First, human-in-the-loop (HITL) checkpoints allocate explicit decision rights who must review, approve, or override algorithmic recommendations at each risk tier. Second, rationale transparency instruments (model registries, versioning, data lineage, and decision logs) ensure that both algorithmic and managerial rationales are captured for post-hoc review. Third, escalation protocols tie actions to risk levels, defining triggers (e.g., out-of-distribution detection, breaches of fairness thresholds, low confidence intervals) that mandate re-routing decisions to higher governance layers (Stix, 2022).

Sustained advantage in AI-enabled management depends on institutionalised learning loops that evolve mental models alongside tooling. Two mechanisms are especially effective. Post-decision reviews (PDRs) compare intended rationales to realised outcomes, auditing both model performance (accuracy, stability, drift) and human judgments (escalations, overrides, trade-offs). Scenario-based drills stress-test decision rules against shocks (demand spikes, regulatory changes, supply disruptions) to reveal brittle assumptions and prompt rule or threshold updates. Together, these practices strengthen system thinking, refresh shared mental models, and narrow the gap between how decisions are supposed to be made and how they are actually made (Chui et al., 2023).

Interpreting the broader organisational implications, the data suggest that hybrid competence is only effective when supported by a coherent decision architecture. Such architecture ensures that AI-assisted decisions remain auditable, reversible, and aligned with organisational risk appetite.

### 5.3 Enablement strategies

Even with sound architecture, execution falters if capability gaps persist. Enablement should target two domains: data literacy and governance fluency. Data literacy programs move beyond tool training to cover data provenance, sampling bias, performance metrics, calibration, and the limits of generalisation. Governance fluency clarifies roles and accountabilities, acceptable-use policies, model lifecycle controls, incident response, and documentation norms (e.g., model cards, datasheets). Organisations can codify these competencies through decision playbooks that standardise prompts, interpretability checklists, override rationales, and escalation pathways by use case.

Concrete interventions include modular microlearning for managers on reading uncertainty bands and interrogating feature importance; simulation-based workshops that rehearse overrides and escalations on realistic cases; and communities of practice where domain experts and data scientists co-curate patterns and pitfalls. For example, a healthcare network can run monthly “decision rounds” where clinicians and analysts jointly review algorithmic recommendations versus clinical outcomes, updating playbooks accordingly. A manufacturing conglomerate might deploy an enterprise model registry with mandatory pre-deployment checklists and post-deployment monitoring dashboards, tying compliance to executive scorecards (Chui et al., 2023). These enablement levers accelerate adoption while aligning with emerging regulatory expectations around transparency, risk management, and human oversight [Regulation (EU) 2024/1689].

Here is a comparison Table 3 of Strategic Thinking vs. Computational Thinking in Management, aligned with Peter Senge’s Five Disciplines:

**Table 3:** Strategic thinking vs. computational thinking in management

Discipline	Strategic thinking	Computational thinking
Systems Thinking	Holistic visioning	Modelling and simulation
Personal Mastery	Self-awareness and growth	Skill optimization via data
Mental Models	Challenging assumptions	Algorithmic reasoning
Shared Vision	Value alignment	Goal quantification
Team Learning	Dialogue and reflection	Collaborative automation

This table illustrates how strategic thinking emphasises human-centric, value-driven approaches, whereas computational thinking focuses on structured, data-driven logic and automation.

Here is a comparative table that includes Peter Senge’s managerial roles (Five Disciplines) and examples of problems that arise when managers mistakenly choose Strategic Thinking or Computational Thinking inappropriately. This Table 4 illustrates where wrong thinking creates risks:

**Table 4:** Roles, thinking types, and mistaken choices.

Discipline	Role	Strategic thinking error	Computational thinking error
Systems Thinking	Planner	Ignores data trends	Over-models without context
Personal Mastery	Leader	Relies only on intuition	Automates personal growth
Mental Models	Analyst	Assumes outdated beliefs	Misses nuance in logic
Shared Vision	Facilitator	Overemphasizes ideals	Quantifies values poorly
Team Learning	Coach	Avoids metrics	Overuses dashboards

Strategic Thinking errors often stem from ignoring data and overvaluing intuition. Computational Thinking errors arise from over-automation and the neglect of human factors. Here are real-world hybrid case studies that illustrate how combining strategic thinking and computational thinking to form a hybrid competence management framework (Table 5).

**Table 5:** Hybrid competence management framework.

Discipline	Hybrid case study
Systems Thinking	Thoughtful grid planning with analytics and stakeholder input
Personal Mastery	AI-powered leadership coaching (e.g., BetterUp)
Mental Models	Scenario planning with AI simulations
Shared Vision	ESG dashboards + employee value surveys
Team Learning	Miro + GPT for collaborative summarization

The integration of strategic and computational thinking across Mintzberg’s 10 managerial roles reveals a cognitive reconfiguration of leadership in AI-augmented governance. Strategic thinking, historically grounded in systems theory, long-term visioning, and normative reasoning, remains indispensable in roles that require ethical discernment, stakeholder alignment, and effective symbolic representation, such as those of the figurehead, leader, and negotiator. These roles demand human-centred judgment, influence-based leadership, and contextual sensitivity that computational logic cannot replicate.

Conversely, computational thinking, characterised by algorithmic reasoning, pattern recognition, and optimisation, has become increasingly salient in roles such as monitor, disseminator, and resource allocator. Here, AI systems enhance managerial capacity by automating data synthesis, forecasting, and decision support. However, the utility of computational augmentation is contingent upon the presence of strategic guardrails. Without human-in-the-loop (HITL) oversight, algorithmic outputs risk misalignment with organisational values, stakeholder expectations, or broader institutional legitimacy.

This duality highlights the need for a hybrid managerial model. In such a model, strategic thinking sets the purpose, boundaries, and ethical constraints, while computational thinking operationalises choices within those parameters. For example, the entrepreneur role benefits from AI-enabled scenario simulations, but it is strategic foresight that determines which futures are desirable or permissible. Similarly, the disturbance handler may rely on risk modelling tools, but must interpret them through the lens of equity, accountability, and public trust.

The implications for governance are profound. AI-augmented decision architectures must incorporate structural safeguards, transparent rationale, escalation protocols, and override thresholds to ensure that managerial authority remains accountable and ethically grounded. These mechanisms reflect the normative imperatives codified in instruments such as the EU Artificial Intelligence Act (Regulation (EU) 2024/1689), which institutionalises human oversight in high-risk decision domains. Moreover, the redefinition of managerial roles in light of cognitive hybridity necessitates new forms of institutional enablement: training in data literacy, governance fluency, and ethical reasoning must be complemented by technical proficiency.

Mintzberg’s framework, when reinterpreted through the lens of strategic–computational hybridity, offers a valuable heuristic for navigating the socio-technical transformations reshaping organisational life. It affirms that managerial legitimacy in the age of AI depends not on the substitution of human judgment, but on its augmentation through principled, transparent, and accountable use of computational tools. This hybrid cognition is not merely a technical adaptation it is a normative evolution in how institutions govern, learn, and lead (Table 6).

**Table 6:** Comparative analysis of managerial roles.

Mintzberg role	Strategic thinking contribution	Computational thinking contribution
Figurehead	Embodies organizational values and culture; symbolic leadership	Limited; ceremonial support via scheduling tools
Leader	Vision-setting, ethical alignment, influence-based leadership	Performance analytics, AI-assisted coaching platforms
Liaison	Coalition-building, stakeholder diplomacy	Network mapping, sentiment analysis
Monitor	Environmental scanning, strategic interpretation	Anomaly detection, dashboard analytics
Disseminator	Narrative framing, internal alignment	Automated reporting, summarization tools
Spokesperson	External representation, strategic messaging	Media analytics, trend forecasting
Entrepreneur	Innovation, scenario planning, adaptive strategy	Simulation modelling, predictive analytics
Disturbance Handler	Crisis management, ethical judgment under uncertainty	Risk modelling, diagnostic tools
Resource Allocator	Strategic trade-offs, long-term investment decisions	Optimization algorithms, budget simulations
Negotiator	Bargaining, power dynamics, stakeholder alignment	Data-driven negotiation prep, outcome modelling

Roles traditionally anchored in strategic thinking, such as Figurehead, Leader, and Negotiator, continue to rely heavily on human judgment, ethical reasoning, and relational intelligence. These roles scored higher on strategic thinking items, particularly in sectors such as healthcare and public-facing retail, where ambiguity and stakeholder sensitivity are prevalent.

Conversely, roles such as Monitor, Disseminator, and Resource Allocator demonstrated more substantial alignment with computational thinking, particularly in the technology and finance sectors. These managers demonstrated higher confidence in AI tools, data-driven decision-making, and pattern recognition, reflecting the operational integration of algorithmic support in routine tasks.

The Entrepreneur and Disturbance Handler roles emerged as hybrid zones, where strategic foresight and computational modelling must coalesce. Managers in these roles reported using AI simulations and risk diagnostics but retained override authority based on ethical thresholds and contextual judgment. This bifurcation underscores the importance of human-in-the-loop (HITL) governance, particularly in high-stakes or ethically complex decisions.

Overall, the data support a hybrid competence model: strategic thinking sets normative boundaries and long-term direction, while computational thinking enhances precision, scalability, and responsiveness. The effectiveness of Mintzberg’s roles in AI-intensive environments depends on the organisation’s ability to institutionalise this cognitive duality through training, decision architecture, and governance protocols. The findings affirm that managerial legitimacy in the digital age is not a function of technological substitution, but of principled augmentation.

Overall, the data suggest that hybrid managerial competence does not arise from a uniform integration of strategic and computational thinking. Instead, hybrid cognition emerges through patterned co-occurrence, shaped by sector, role, organisational maturity, and task demands. Strategic reasoning continues to set the normative and interpretive boundaries of managerial work, while computational thinking provides analytic depth, scale, and precision. Managerial legitimacy in the age of AI therefore depends not on technological substitution but on the principled augmentation of human judgment supported by decision architecture, capability building, and context-sensitive coordination of cognitive modes.

## 6 Conclusion

In conclusion, this study advances a hybrid cognitive framework for managerial decision-making in AI-augmented environments, grounded in Peter Senge’s Five Disciplines. It responds to the research question of how strategic and computational thinking interact across Mintzberg’s ten managerial roles, and tests the hypothesis that managerial effectiveness is maximised when these paradigms are integrated. The findings confirm that strategic thinking anchored in systems thinking, ethical foresight, and shared vision remains essential for roles requiring contextual judgment and stakeholder alignment. Computational thinking, which enhances mental models and team learning, supports roles focused on data synthesis, optimisation, and operational precision. The integration of both paradigms enables managers to navigate complexity, uphold institutional legitimacy, and reconcile normative goals with analytical rigour. The findings indicate that strategic thinking rooted in systems thinking, ethical foresight, and shared vision remains essential in roles requiring contextual interpretation, stakeholder alignment, and normative judgment. Conversely, computational thinking plays a more prominent role in tasks involving data synthesis, optimisation, pattern recognition, and operational precision. The coexistence of these modes allows managers to maintain situational awareness while leveraging algorithmic support where it adds the most value. Ultimately, the study affirms that managerial legitimacy in the digital age depends not on the substitution of human judgment, but on its principled augmentation through transparent, accountable, and ethically aligned use of computational tools. This framework is a concrete micro-level response to the opportunities and tensions of the management competencies.

A key limitation of this study are that the study is exploratory and based on a modest sector-level sample, with short three-item indices that function as descriptive proxies rather than validated scales. Findings rely on self-reported questionnaire data and do not include outcome variables, limiting causal inference. A synthetic simulation is used solely for conceptual illustration and does not constitute empirical evidence.

A promising future research direction is the development of micro-level KPI dashboards that track behavioural signals such as how managers interrogate AI recommendations, how often they apply human-in-the-loop controls, and how effectively they adjust their mental models in response to feedback. Moving from conceptual framing to quantitative, KPI-linked validation is essential for understanding the practical value of hybrid strategic–computational thinking. This line of research can provide evidence-based guidance for leaders, AI governance councils, and policymakers seeking to optimise managerial performance, accountability structures, and organisational resilience in AI-intensive environments.

## Data Availability

The original contributions presented in the study are included in the article/Supplementary material, further inquiries can be directed to the corresponding author.
